# Visualization of BRI1 and SERK3/BAK1 Nanoclusters in *Arabidopsis* Roots

**DOI:** 10.1371/journal.pone.0169905

**Published:** 2017-01-23

**Authors:** Stefan J. Hutten, Danny S. Hamers, Marije Aan den Toorn, Wilma van Esse, Antsje Nolles, Christoph A. Bücherl, Sacco C. de Vries, Johannes Hohlbein, Jan Willem Borst

**Affiliations:** 1 Laboratory of Biochemistry, Wageningen University & Research, Stippeneng 4, WE Wageningen, The Netherlands; 2 Laboratory of Biophysics, Wageningen University & Research, Stippeneng 4, WE Wageningen, The Netherlands; 3 Microspectroscopy Centre, Wageningen University & Research, Stippeneng 4, WE Wageningen, The Netherlands; University of Wisconsin Madison, UNITED STATES

## Abstract

Brassinosteroids (BRs) are plant hormones that are perceived at the plasma membrane (PM) by the ligand binding receptor BRASSINOSTEROID-INSENSITIVE1 (BRI1) and the co-receptor SOMATIC EMBRYOGENESIS RECEPTOR LIKE KINASE 3/BRI1 ASSOCIATED KINASE 1 (SERK3/BAK1). To visualize BRI1-GFP and SERK3/BAK1-mCherry in the plane of the PM, variable-angle epifluorescence microscopy (VAEM) was employed, which allows selective illumination of a thin surface layer. VAEM revealed an inhomogeneous distribution of BRI1-GFP and SERK3/BAK1-mCherry at the PM, which we attribute to the presence of distinct nanoclusters. Neither the BRI1 nor the SERK3/BAK1 nanocluster density is affected by depletion of endogenous ligands or application of exogenous ligands. To reveal interacting populations of receptor complexes, we utilized selective-surface observation—fluorescence lifetime imaging microscopy (SSO-FLIM) for the detection of Förster resonance energy transfer (FRET). Using this approach, we observed hetero-oligomerisation of BRI1 and SERK3 in the nanoclusters, which did not change upon depletion of endogenous ligand or signal activation. Upon ligand application, however, the number of BRI1-SERK3 /BAK1 hetero-oligomers was reduced, possibly due to endocytosis of active signalling units of BRI1-SERK3/BAK1 residing in the PM. We propose that formation of nanoclusters in the plant PM is subjected to biophysical restraints, while the stoichiometry of receptors inside these nanoclusters is variable and important for signal transduction.

## Introduction

Brassinosteroids (BRs) are plant steroid hormones that regulate cellular expansion, differentiation and proliferation [1]. The brassinosteroid signalling pathway starts at the plasma membrane (PM), where BRs bind to the island domain in the extracellular part of the leucine-rich-repeat receptor like kinase (LRR-RLK) BRASSINOSTEROID-INSENSITIVE1 (BRI1). Ligand binding induces phosphorylation and subsequent disassociation of the inhibitor protein BRI1 KINASE INHIBITOR 1 (BKI1) from the cytoplasmic kinase domain of BRI1 [2, 3]. BKI1 prevents BRI1 from interacting with its co-receptor SERK3/BAK1 (SOMATIC EMBRYOGENESIS RECEPTOR KINASE 3/ BRI1 ASSOCIATED KINASE 1) [2]. Hetero-oligomerization of BRI1 and SERK3/BAK1 results in sequential trans phosphorylation events on their cytoplasmic kinase domains [4], which is a prerequisite for successful BR signal transduction [5]. Trans phosphorylation between SERK3/BAK1 and BRI1 leads to full activation of the signalling pathway resulting in phosphorylation of downstream signalling components [6, 7]. The signal is further relayed to the transcription factors BZR1 and BES1, resulting in regulated expression of BR- responsive genes. Recent investigations suggest that hetero-oligomers of BRI1 and SERK3/BAK1 are, at least partially, preformed in absence of ligand and form a functional unit able to perceive BRs and initiate downstream signalling [8]. Extracellular domain interactions between SERK1, a highly homologous family member of SERK3/BAK1, and BRI1 are ligand dependent [9], suggesting that domains such as the transmembrane domain or other cytoplasmic domains are essential for the observed ligand independent hetero-oligomerisation.

BRI1 and SERK3/BAK1 have a fundamental role in BR signalling and regulation of BR-related developmental processes in root and shoot [10, 11]. Although there are indications of endosomal BR signalling [12], the initial recognition of BRs and activation of the receptor complex via ligand binding occurs at the PM [13]. The PM is a highly organized lipid bilayer interspersed with proteins, of which most of them show restricted diffusion through the lipid bilayer and even clear inhomogeneous patterning across the PM [14 and references therein]. Diffusion of proteins in the PM can be restricted via the cortical cytoskeleton, protein ‘crowding’, interaction between membrane components or heterogeneity in membrane composition and state [14]. In *Arabidopsis*, the lateral movement of PM localized proteins is further restricted by the presence of the cell wall, although not necessarily due to direct interactions [15]. As a result of these restrictions, protein distribution across the membrane is likely to lead to cluster formation. In animal cells, the presence of so-called nanoclusters of receptor proteins in the PM has been established [16] and is thought to be essential for signal transduction. In addition, internalization of receptors upon ligand-binding- through endocytosis has been reported to take place via specific endosomal locations [17] and by relocalization within the PM itself [18].

Recently, the plant receptor kinase BRI1 was found to localize in nanoclusters or membrane microdomains in the PM [19]. Upon BR stimulation, an increased colocalization of BRI1-GFP with a membrane microdomain marker protein (AtFlot1-mCherry) was observed. This partitioning of BRI1 into microdomains has been suggested to be essential for BR signalling.

Here, we investigate the PM distribution of BRI1 and SERK3/BAK1 in live *Arabidopsis thaliana* epidermal root cells using variable-angle epifluoresence microscopy (VAEM) [20] and fluorescence lifetime imaging microscopy (FLIM) for the detection of Förster resonance energy transfer (FRET). Different lines of BRI1-GFP were used in this study of which BRI1-GFP line 1 has endogenous protein expression levels whereas BRI1-GFP line 2 showed a threefold higher expression [21]. Our results show an inhomogeneous distribution of BRI1 and SERK3/BAK1 depicted by cluster formation across the membrane. The cluster density is not altered by activating the signalling complex; neither by over-expression of the receptor nor by changing endocytosis rate of the receptor. To characterize BRI1-SERK3/BAK1 hetero-oligomers, we performed FRET in combination with FLIM in the plane of the PM of root epidermal cells adopted as Selective Surface Observation—FLIM (SSO-FRET-FLIM). Using this approach, we revealed that BRI1-SERK3/BAK1 hetero-oligomers are present as the active ligand perception units within nanoclusters in epidermal cells of *Arabidopsis* roots.

## Results

### BRI1 and SERK3 are present in plasma membrane nanoclusters

To investigate the distribution of BRI1-GFP and SERK3/BAK1-mCherry at the PM we imaged these receptors using VAEM. Both receptors form hetero-oligomeric complexes required for signal transduction and are part of the same signalling pathway [2, 8].

**Fig 1:**
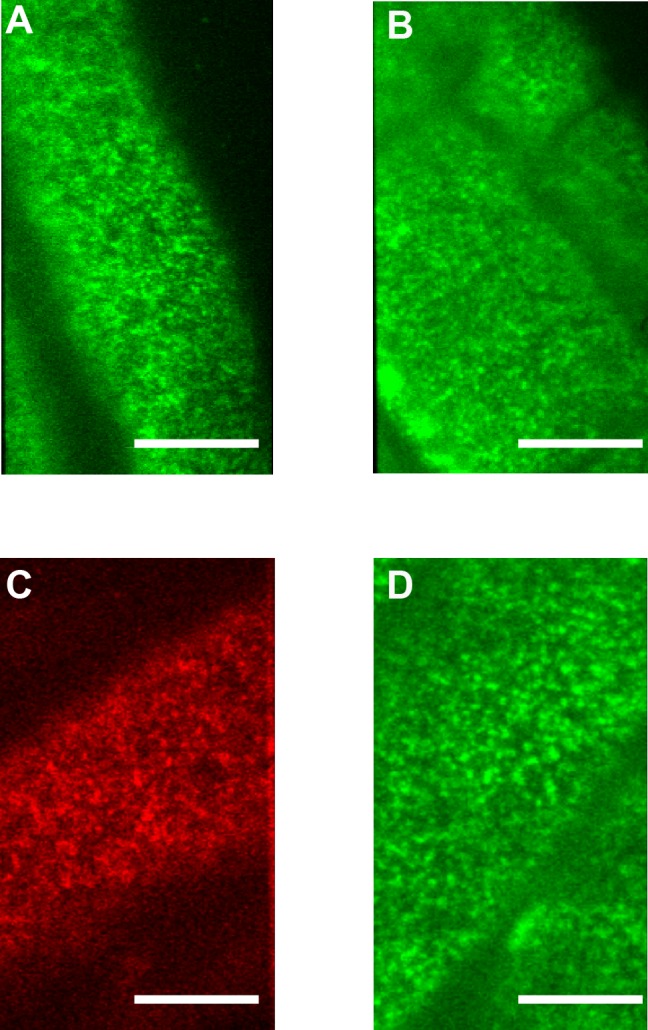
VAEM reveals a heterogeneous distribution of BRI1-GFP and SERK3-mCherry in the PM. Typical VAEM images of live root epidermal cells of 6 day old *A*. *thaliana* seedlings showing PM distribution of (**A**) BRI-GFP line1, (**B**) BRI1-GFP line 2 and (**C**) SERK3-mCherry, (**D**) BRI1-GFP in a *serk1serk3* mutant plant. Images taken are of epidermal cells in the early elongation zone. Scale bars represent 10 μm.

VAEM showed that neither BRI1 nor SERK3/BAK1 is homogenously distributed. Furthermore, a clear punctuated pattern was seen for both receptors (Fig 1), in contrast to the homogeneous distribution of PM-marker LT16-B (S1 Fig), an integral membrane protein [22]. Similar receptor distributions in animal cells are referred to as nanoclusters [23], a term that we will employ here as well. Nanoclusters of clearly variable fluorescence intensities were observed with an average size of approximately 5 pixels per cluster, which corresponds to a diameter of 300–500 nm. These nanoclusters were observed for both the main receptor BRI1 and the coreceptor SERK3/BAK1 (S2–S6 Movies; a step by step guide for image analysis is provided in S2 Fig). The receptors can be visualized by VAEM in the epidermal root cells of the elongation zone, an area where BR signalling is reported to be active [10, 24]. The root meristem zone itself cannot be visualized due to curvature of the root, which causes the epidermal cell layer to be outside the critical range for VAEM. Our VAEM setup was capable of visualizing endosomes and other internal membrane compartments demonstrated by imaging of the ARA6/Rab F1-mRFP marker [25] (S1 Fig). Also, the ER marker WAVE6-mCherry [26] was visualized using VAEM. In plant cells, the ER forms a net-like basket occupying the cortical space just below the plasma membrane. Time-lapse imaging revealed that the ER is a mobile and dynamic organelle (S1 Movie). We used additional markers of Golgi stacks (WAVE18-mRFP, [26], Trans Golgi Network (VHAa1-mRFP, [27]) and retrograde trafficking (ARA7/Rab F2b, [25, 28] to track intracellular membrane compartments in conjunction with the PM of *Arabidopsis* root epidermal cells (S1 Fig).

Endosomal vesicles on the cytoplasmic side of the PM were clearly visible for both BRI1 and SERK3/BAK1, with a higher number of BRI1-containing vesicles compared to SERK3/BAK1. Especially BRI1-GFP line 2, which has approximately 3x higher expression of BRI1-GFP compared to wild type, shows a large number of fluorescent endosomal compartments, which are reminiscent of LE/EE compartments (S2 and S3 Movies). Interestingly, only a small number of nanoclusters containing BRI1-GFP were seen to disappear from the PM (S4 Movie) even in the presence of brassinazole (BRZ), a potent brassinosteroid synthesis inhibitor [29]. Upon application of BL, we did not observe a difference in the rate of internalization of nanoclusters from the PM by endocytosis. Recently, the existence of ligand-independent BRI1 endocytosis was discussed in reference [30].

Brassinosteroid signalling is dependent on both the presence of the main ligand binding receptor BRI1 and the SERK co-receptors [5]. In the root, the active signalling complex consists of BRI1 with SERK1 and/or SERK3/BAK1 [31, 32]. We recently showed that BRI1 and SERK3/BAK1 co-localize in the PM and that a minor amount of BRI1 and SERK3/BAK1 receptors is already present in preformed complexes [8]. To test whether the co-receptors influence the distribution of the main receptor in the PM, the BRI1-GFP line 2 was crossed into a *serk1serk3* mutant background. VAEM showed that in the absence of both co-receptors the distribution of the main ligand binding receptor and the overall fluorescence intensity (Fig 1D) are barely affected compared to BRI1-GFP line 2 in wild-type roots. Roots of *serk1serk3* double mutants are almost completely insensitive to BL [33], indicating that no active signalling complexes are formed. Our results thus imply that SERK co-receptors do not participate in maintaining the PM distribution of BRI1 in epidermal cells of *Arabidopsis* roots.

By combining the BRI1 and SERK3/BAK1 PM receptor density data from [21] with the number of nanoclusters per μm^2^ PM determined in this work, we estimated the number of receptors present in each nanocluster (Table 1). In this analysis, we assumed perfect maturation efficiency of fluorescent proteins leading to a possible underestimation of receptor quantities, but still provide information between expression levels between different lines. For example, at near-endogenous receptor levels we estimated that at least 6 fluorescent BRI1 receptors are present in each nanocluster. Intriguingly, while overexpression of BRI1-GFP resulted in about a three-fold increase in PM receptor density [21], there is no concomitant increase in nanocluster density. In fact, the density of BRI1 nanoclusters appeared to be even slightly lower in cells of the overexpression line (Table 1). Two explanations are conceivable: first, either the nanoclusters harbouring PM located receptors can accommodate variable numbers or, second, a larger number of receptors is distributed more uniformly outside of the PM nanocluster domains upon overexpression. In the SERK3-GFP line about two-fold more nanoclusters were present compared to BRI1-GFP line 1, of which each contained at least two receptors. A similar calculation for the SERK3/BAK1-mCherry line resulted in about 1 ± 1 receptors per nanocluster. The number of receptors per nanocluster is in line with estimates based on single-molecule photo bleaching analysis (S3 Fig).

**Table 1:** Quantification of the number of receptors per nanocluster.

Plant line	receptors per μm^2^ at the PM*	nanoclusters per μm^2^ at the PM	Number of receptors per nanocluster
BRI-GFP line 1	12 ± 1	2 ± 0.4 (n = 9)	6 ± 1
BRI-GFP line 2	34 ± 3	1.5 ± 0.4 (n = 9)	22 ± 4
SERK3-GFP	5 ± 1	3 ± 0.4 (n = 11)	2 ± 1

The average number of receptors (second column taken from reference [21]) and the number of nanoclusters per μm^2^ (third column) allows calculating the average number of receptors per nanocluster (fourth column). Numbers are given with their respective standard error of the mean (SEM). For each experiment, three different roots were recorded using confocal imaging (n = 9). n = number of individual cells. Values are given ±SEM.

* data from [21]

A third plant receptor was investigated to rule out that the observed nanocluster distribution pattern was an inherent property of only these two receptors. BIR3 (BAK1-interacting receptor kinase 3) is an abundant PM receptor-like kinase for which the related members BIR1 [34] and BIR2 [35] have been implicated as stabilizing components of PM receptor complexes involved in pathogen triggered immunity. Similar to BRI1 and SERK3, BIR3 is distributed heterogeneously in the PM including punctuated pattern, suggesting that a distribution into nanoclusters is a common configuration for plant membrane receptors (S4 Fig). Other PM proteins such as remorins and flottilins also showed nanocluster arrangements harbouring about 0.1–1.3 domains per μm ^2^ [36].

### BRI1 PM-distribution is not altered upon signal activation or absence of endogenous ligands

In order to determine whether BRI1 and SERK3/BAK1 receptor distribution across the PM is affected by activation of the BR signalling pathway, seedlings were first depleted of endogenous ligands by incubation with BRZ. Prior to VAEM, the BL depleted seedlings were incubated with 1 μM BL, which is the biologically most active brassinosteroid, for 1 h. This treatment is routinely used to fully activate the BR signalling pathway and optimized to achieve a near maximal receptor-ligand occupancy [33]. Interestingly, under these conditions of full activation, only a minor amount of BRI1-GFP and SERK3/BAK1-mCherry was previously found to interact [8]. It was therefore of great interest to test whether changes in the distribution or number of nanoclusters would be visible upon full activation of the signalling pathway. Surprisingly, we could not detect any significant change in the number of clusters per μm^2^ at the PM or cluster size for both BRI1-GFP and SERK3/BAK1-mCherry between the BRZ treated roots and the BRZ+BL treated roots (Table 2). Roots expressing SERK3/BAK1-mCherry showed a non-significant increase in the number of clusters upon BRZ treatment. BRI1-GFP distribution was also not affected in the brassinosteroid synthesis mutant *det*2 (S5 Fig), which contains less than 10% of the normal WT levels of brassinosteroids [37].

**Table 2:** Quantification of clusters/ μm^2^ and cluster size of BRI1 and SERK3.

		BRZ	BL after BRZ pre-treatment	No treatment
**BRI1-GFPline 1**	clusters/ μm^2^	0.55 ± 0.13	0.49 ± 0.08	0.59 ± 0.1
	cluster size (μm^2^)	0.29 ± 0.04	0.31 ± 0.04	0.29 ± 0.02
**BRI1-GFP line 2**	clusters/ μm^2^	0.54± 0.14	0.60 ± 0.14	0.59 ± 0.14
	cluster size (μm^2^)	0.45 ± 0.06	0.49 ± 0.06	0.41 ± 0.05
**SERK3-mCherry**	clusters/ μm^2^	1.05 ± 0.19	0.75 ± 0.14	0.83 ± 0.11
	cluster size (μm^2^)	0.12 ± 0.02	0.08 ± 0.03	0.13 ± 0.02

Quantification of nanocluster density and size of BRI1-GFP and SERK3/BAK1-mCherry of 5 day old *Arabidopsis* seedlings showing the average number of nanoclusters per μm^2^ and average cluster size in μm^2^ upon different treatments using VAEM. 5 μM BRZ was added to growth media for 3 days prior to ligand application. Ligand application was accomplished by incubation of seedlings with 1 μM BL for 1h. All experiments were performed twice; three images were collected for three seedlings per experiment (n = 9). Numbers are given with their respective pooled standard error of the mean (SEM).

### BRI1 and SERK3/BAK1 mobility in the PM investigated using FRAP

**Fig 2:**
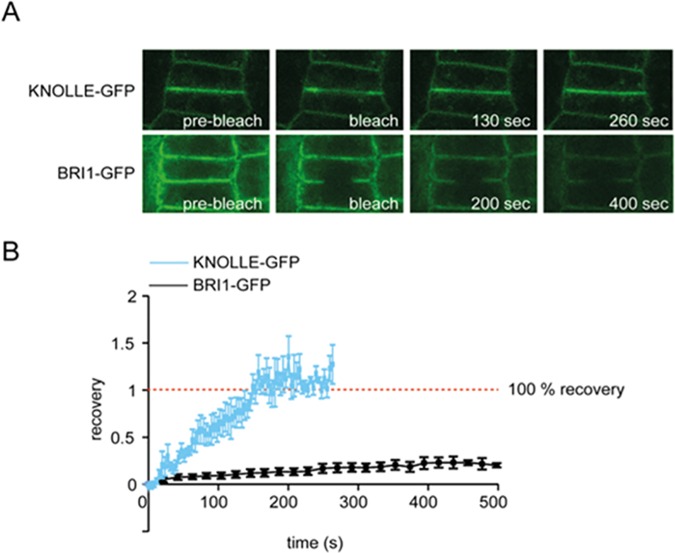
FRAP analysis of BRI1-GFP and KNOLLE-GFP in epidermal cells of *Arabidopsis* roots. (**A**) Typical images of FRAP experiment prior and post bleach pulse of KNOLLE-GFP and BRI1-GFP. (**B**) Recovery-curves of KNOLLE-GFP (blue line) and BRI1-GFP (black line) in epidermal cells in the root meristem. Around 200 s after bleaching, fluorescence intensity of KNOLLE-GFP at the PM is fully restored. No such recovery is observed for BRI1-GFP. For KNOLLE-GFP n = 7, for BRI1-GFP n = 15, measured in independent replicas, error bars indicate standard error of means (SEM).

Using VAEM, we observed different mobility states, ranging from static for BRI1 and SERK 3/BAK1 to rapid directional movement ER marker (S1–S6 Movies). In order to characterize the mobility of BRI1 and SERK3/BAK1 receptors in the PM, we performed fluorescence recovery after photobleaching (FRAP) on fluorescently labelled receptors expressed in Arabidopsis roots. In the elongation zone of *Arabidopsis* root cells, BRI1-GFP and SERK3/BAK1-GFP showed a slow diffusion (below 0.1 μm^2^*s^-1^) and a mobile fraction of 35 and 60% for BRI1 and SERK3/BAK1, respectively. These values were at the lower end of 0.1–1.0 μm^2^*s^-1^, reported for free lateral diffusion of a PM receptor [15, 19] and of control measurement (Fig 2). In meristem cells, the diffusion coefficients were even lower, rendering both receptors virtually immobile (Table 3 and S6 Fig). Further analysis employing a one-dimensional Gaussian fit of the fluorescence intensity over the cross section of the PM suggests that most of the observed mobility in the elongation zone is due to replenishment from internal receptor pools (S7 Fig). In a similar study by Wang and coworkers [19], BRI1-GFP was expressed under its native promoter in Col 0 background and a diffusion coefficient of 8.8x10^-3^ μm^2^*s^-1^ was found, which is in similar range to our observations in meristem cells. Taken together, our VAEM and FRAP data suggest that both BRI1 and SERK3/BAK1 receptors are distributed in PM nanoclusters, which are largely immobile and contain only a limited number of BRI1 and SERK3/BAK1 receptors.

**Table 3:** Mobile fractions and diffusion coefficients of BRI1 and SERK3/BAK1.

	Tissue	Mf (%)	D μm^2^*s^-1^	*n*
**BRI1-GFP**	Meristem	28 ± 2	0.003 ± 0.001	15
	Elongation zone	35 ± 3	0.04 ± 0.02	15
**SERK3/BAK1-GFP**	Meristem	78 ± 3	0.003 ± 0.001	5
	Elongation zone	58 ± 2	0.08 ± 0.02	5

n = number of individual measurements. Mf = mobile fraction. D = diffusion coefficient. Values are given ± SEM

### BRI1 and SERK3/BAK1 distribution is not influenced by various inhibitors

All three integral membrane receptors investigated in this study, BRI1, SERK3/BAK1 and BIR3, showed a similar distribution of nanoclusters. In addition, BRI1 and SERK3/BAK1 nanocluster distribution was not affected upon ligand induced activation. To investigate the underlying mechanism regulating membrane distribution, we treated *Arabidopsis* roots with various inhibitors of the endocytic, cytoskeleton or biosynthetic pathways. Treatment of BRI1-GFP and SERK3/BAK1-mCherry seedlings with cycloheximide, latrunculin B or brefeldin A either alone or in combination with BRZ or with BRZ and BL did not result in any consistent change in distribution of either receptor (data not shown).

### Receptor interactions visualized by selective-surface observation FRET-FLIM

**Fig 3:**
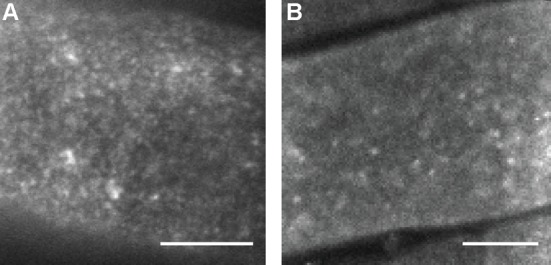
BRI1-GFP nanoclusters imaged using VAEM and SSO-confocal imaging. BRI1-GFP line 2 expressed in root epidermal cells imaged using VAEM (A) or SSO-confocal imaging (B). Both imaging modalities show similar nanocluster distributions. Scale bars represent 10 μm.

A limitation of VAEM is the inability to report on the interaction between BRI1-SERK3/BAK1 hetero-oligomers. We therefore performed SSO FRET-FLIM by focussing the confocal spot at the PM of root epidermal cells. As expected, fluorescence intensity images obtained with this approach (Fig 3B) revealed a similar distribution of BRI1-GFP nanoclusters as found using VAEM (Fig 3A). Notably, VAEM can acquire single images within a few hundred milliseconds, whereas a SSO confocal image requires an acquisition time between one and two minutes.

**Fig 4:**
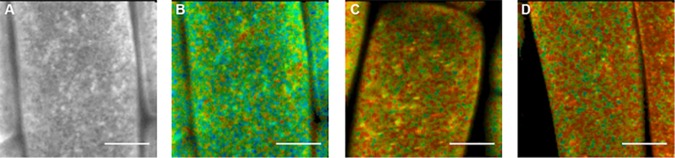
SSO-FRET-FLIM of BRI1-GFP and BRI1-GFP/SERK3/BAK1-mCherry in absence and presence of BL. (**A**) SSO fluorescence intensity image of BRI1-GFP expressed in root epidermal cells pretreated with 50 μM BRZ (**B**) SSO fluorescence lifetime image of BRI1-GFP expressed in root epidermal cells pretreated with 50 μM BRZ (**C**) SSO fluorescence lifetime image of BRI1-GFP/SERK3/BAK1-mCherry expressed in root epidermal cells pretreated with 50 μM BRZ. (**D**) SSO fluorescence lifetime image of BRI1-GFP/SERK3/BAK1-mCherry expressed in root epidermal cells pretreated with 50 μM BRZ and subsequent incubation with 1 μM BL for 1 hour. The color bar represents the false color code for fluorescence lifetime (*τ*) distribution. Scale bar represents 10 μm.

The spatial distribution of fluorescence lifetimes of BRI1-GFP nanoclusters is not homogeneous even though the standard deviation of all samples analysed is small (*τ* = 2402 ± 33 ps, Table 4). At present, it is not clear whether this is due to small local variations in the immediate environment of the receptors inside nanoclusters or whether it is due to technical limitations, such as the precision by which the confocal volume can be positioned with respect to the plane of the PM. In root cells expressing both BRI1-GFP and SERK3-mCherry, a small number of nanoclusters showed reduced fluorescence lifetimes (*τ* = 2227 ± 134 ps, Table 4). This observation indicated that a few nanoclusters located in the root epidermal cell PM contain both receptors in close proximity, leading to a detectable FRET signal (Fig 4). BRZ treatment followed by application of BL showed only a minor reduction in overall fluorescence lifetimes of BRI1-GFP or BRI1-GFP/SERK3/BAK1-mCherry expressing root cells as determined by SSO FRET-FLIM (*τ* = 2241 ±103 ps, Table 4). This finding is in full accordance with recently published data using conventional FRET-FLIM, showing only a small increase in interaction between BRI1 and SERK3/BAK1 upon full activation of the signalling pathway [8]. No significant change of donor fluorescence lifetimes was observed in roots depleted of endogenous BL or stimulated with BL (Table 4) independent of analysis conditions (see materials and methods) suggesting that that no significant changes in the size or composition of nanoclusters occurred upon ligand application.

**Table 4:** Quantitative analysis of SSO FRET-FLIM measurements performed in planta on roots of 5 day old *Arabidopsis* roots expressing BRI1-GFP or BRI1-GFP -SERK3/BAK1-mCherry.

	Treatment	*τ* (ps)	FRET (%)	IPS (%)	*n*
**BRI1-GFP**	BRZ	2402 ± 33	0	0	25
**BRI1-GFP + SERK3/BAK1-mCherry**	BRZ	2227 ± 134^a^	7	15	32
**BRI1-GFP + SERK3/BAK1-mCherry**	BRZ+BL	2241 ± 103^a^	7	10	27

^a^ The mean difference is significant at the (p<0.001) when compared to the donor only sample (BRI1-GFP).

All seedlings were depleted of endogenous brassinosteroids with BRZ and treated with 1 μM BL. *τ* represents the average fluorescence lifetime of the GFP in picoseconds ± SEM. IPS represents the percentage of interacting pixels, and *n* the number of fluorescence lifetime images analysed.

We recently introduced the concept of interaction pixels (IPS) to obtain a more quantitative description of the occurrence of FRET detected by FLIM [8]. Briefly, this method determines the number of pixels in an image that have sufficiently high photon counts as well as a strongly reduced fluorescence lifetime and reports these as a percentage of the total number of pixels. Using IPS in conventional FRET-FLIM, we previously showed that upon BL stimulation in BRZ pre-treated BRI1-GFP/SERK3/BAK1-mCherry expressing roots the IPS increased from about 8% to 13% [8].

Using the same analysis method for our SSO FRET-FLIM results, we determined an IPS of 15% in ligand-depleted roots (Table 4). This percentage is about two-fold higher compared to the 8% found previously using conventional FRET-FLIM [8]. The increase of IPS in SSO FRET-FLIM is possibly due to imaging a larger area of PM located receptors oriented perpendicular to the focal plane. Surprisingly, ligand application resulted in a reduction in the percentage of IPS to about 10% (Table 4) instead of the increase noted earlier [8]. A plausible explanation for this discrepancy can be found in movies of root cells recorded either in VAEM or in SSO mode. During active signalling, occasionally nanoclusters were seen to be endocytosed and thus rapidly disappearing from the PM (S2 Movie). Given the fact that there is no overall change in the number of BRI1 or SERK3/BAK1 containing nanoclusters in the PM after BL ligand application, we conclude that the reduction in IPS is mainly due to endocytosis of nanoclusters containing interacting BRI1 and SERK3/BAK1 receptors.

**Fig 5:**
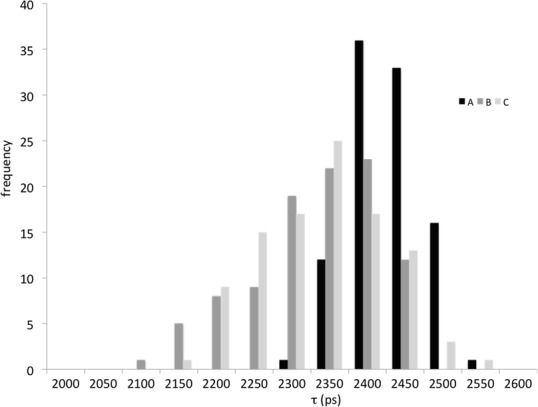
Fluorescence lifetime distribution of high intensity nanoclusters of BRI1-GFP and BRI1-GFP- SERK3/BAK1-mCherry in the absence and presence of BL. Fluorescence lifetime distribution plots of nanoclusters with fluorescence intensities above 2000 photons per pixel. (**A**) Fluorescence lifetime distribution of BRI1-GFP nanoclusters pretreated with 50 μM BRZ (n = 91). (**B**) Fluorescence lifetime distribution of BRI1-GFP/SERK3-mCherry nanoclusters after treatment with 50 μM BRZ (n = 126). (**C**) Fluorescence lifetime distribution of BRI1-GFP-SERK3/BAK1-mCherry nanoclusters pretreated with 50 μM BRZ followed by application of 1 μM BL for 1 hour (n = 95). The data were obtained from 3 independent series of experiments.

We evaluated a series of individual nanoclusters to determine whether the fluorescence lifetimes of individual bright nanoclusters changed during active BR signalling, possibly reflecting a change in the composition of interacting and non-interacting BRI1 and SERK3/BAK1 pairs within a cluster (Fig 5). No significant changes were observed upon ligand application, suggesting that the composition per nanocluster remains unchanged. There also does not seem to be any correlation between the intensity of the nanoclusters and the reduction of donor fluorescence lifetime.

## Discussion

We visualised the distribution of BRI1, SERK3/BAK1 and BIR3 in the PM of *Arabidopsis* root cells using variable-angle epifluorescence microscopy (VAEM) and showed that these receptors are forming nanoclusters. In addition, these nanoclusters contain hetero-oligomers of BRI1-GFP and SERK3/BAK1-mCherry receptors as demonstrated using SSO FRET-FLIM.

Cluster formation of receptors in general was reported earlier. In plants, protein clusters are found for RbohH and PIN proteins [38, 39]. In mammalian cells, the Epidermal Growth Factor Receptor (EGFR) is present in oligomeric clusters in the membrane [23]. These clusters consist of approximately two receptors in an unstimulated situation, which is in the same range we determined for BRI1 and its coreceptor SERK3/BAK1. For EGFR, the nanocluster distribution is thought to be coupled to the biological activity of the receptor [40], and doubling in the number of receptors per cluster is observed upon ligand binding (from ~2 to ~4 receptors per nanocluster; [23]). In contrast, we observed no change in nanocluster distribution for BRI1 or SERK3/BAK1 upon ligand application or depletion of endogenous ligand. Our observation of nanoclusters in plant cells are supported by the work of Jarsch and coworkers [36], who identified membrane structures in the form of microdomains for 20 different plasma membrane localised proteins predominantly belonging to the Remorin protein family. We therefore suggest that the formation of microdomains or nanoclusters is a general feature of plant transmembrane receptors.

Receptors that are arranged in nanoclusters are considered to be part of larger arrangements of signalling proteins [23], where the stoichiometry between different components can be altered without affecting the arrangement [41]. We made similar observations after we compared the PM of two BRI1-GFP lines that differed about 3-fold in receptor density while retaining a similar nanocluster density. Changing the receptor stoichiometry within confinements of nanoclusters could be a mechanism of plant cells to regulate signalling output, especially in the situation of SERK3/BAK1, which is part of different signalling complexes in the same cell [11, 42].

Our FRAP analysis revealed that BRI1-GFP is largely immobile showing a mobile fraction of only 28 ± 2% with a diffusion coefficient of approximately (3.0 ± 1.0) 10^−3^ μm^2^*s^-1^ in meristematic *Arabidopsis* root cells. This is conjunction with the work of Wang and coworkers in which a diffusion coefficient of BRI1-GFP of (8.8 ± 0.6) 10^−3^ μm^2^*s^-1^ was found [19]. As in the case of EGFR, animal receptor nanoclusters are thought to be confined by the cortical actin filament network and cholesterol rich domains [43]. However, for BRI1 and SERK3/BAK1, treatment with Latrunculin B, which is an actin depolymerising agent, did not lead to significant changes in nanocluster distribution. Lateral diffusion of plant receptors is restricted by the presence of the plant cell wall [15]. Restricted diffusion of receptor proteins due to physical barriers such as the underlying cytoskeleton or the cell wall of plant cells could induce clustering of membrane proteins [14]. The classical model for PM receptor activation assumes ligand-induced endocytosis which could imply removal of the entire nanocluster from the PM or a change in their stoichiometry.

Given the observed restrictions in lateral movement shown by our FRAP analysis, it is unlikely that BRI1 nanoclusters are formed after arrival of the proteins at the PM. At present, it is unknown where plant PM receptor nanocluster assembly takes place. One way in which this could be accomplished is via the preformation of higher order signalling complexes, inserted in their respective position in the membrane as a fully assembled unit. Preformation of complexes has been observed for BRI1 and SERK3/BAK1 [8], corroborating this idea. Future research is needed to address the question whether these proteins are indeed inserted in the membrane together, or whether minor mobility within the confinements of the clusters is sufficient to form receptor complexes after insertion in the membrane.

VAEM allows for selective illumination of a thin surface layer and can be used to visualise the plant PM and intracellular membrane compartments in close proximity to the PM, but does not provide spatial information of BRI1-GFP and SERK3/BAK1-mCherry complexes within the nanoclusters. We therefore developed SSO-FRET FLIM as a novel method to show that BRI1-GFP and SERK3/BAK1-mCherry are present in nanoclusters as hetero-oligomers. We observed a reduction of fluorescence lifetime when comparing BL treated BRI1-GFP/SERK3/BAK1-mCherry *Arabidopsis* roots with donor only samples. Furthermore, SSO-FRET FLIM showed no change of the interaction distribution of BRI1-GFP and SERK3/BAK1-mCherry in nanoclusters in the absence or presence of ligand (see Fig 5). The numerical evaluation of IPS significantly reduced fluorescence lifetimes revealing a reduction from 15% to 10% in the number of hetero-oligomers of BRI1-GFP and SERK3/BAK1-mCherry upon signal activation. This reduction in the percentage of IPS is in contradiction with the earlier observed increase of IPS using conventional FRET-FLIM [8]. During active signalling, occasionally nanoclusters were seen to be endocytosed and thus rapidly disappearing from the PM (S2 Movie), similar to what has been observed by Wang et al. 2015 [19]. Using conventional FRET-FLIM, the endocytosed complexes of BRI1-SERK3/BAK1 nanoclusters remain in the focal imaging plane and are taken into account in the IPS analysis. We further conclude that the observed nanoclusters containing interacting oligomers of BRI1 and SERK3/BAK1 represent preformed receptor complexes that upon ligand application do not change in composition and are subsequently endocytosed after signal activation.

We observed nanoclusters with varying fluorescence lifetimes indicating either changes in microenvironment of BRI1-GFP or interaction between BRI1 and SERK3/BAK1. We propose that the varying fluorescence lifetimes in the observed nanoclusters are a result of different receptor stoichiometries. When considering the scale on which proteins interact and the observed cluster size, it has to be noted that the observed nanoclusters most likely contain multiple receptor pairs and other proteins. It remains unknown if the decrease in IPS is due to a conformational change of the receptor complex or due to endocytosis of active signalling complexes.

Further investigation of underlying organelles could provide interesting answers into the dynamics of BR signal activation within the complex confinements of the plant PM and cell wall. Plant receptor distribution at the PM could be a result of intrinsic properties of the PM mediated by actin [44] or microtubule structures. Such clustering might be influenced by endocytosis or incorporation rates of proteins in the PM through contact with the underlying cortical ER. The existence of nanoclusters can play a gate way function in regulating signalling responses, limiting effects of minor small increases in ligand availability and establishing a threshold concentration for signal activation [45, 46]. This way of signalling might be less susceptible to variations in internal and external influences thereby increasing signalling fidelity. In accordance to this trend exogenous application of BRs results in a hyperbolic root growth response curve [21].

The receptor clusters observed with VAEM showed a diameter of 300–500 nm, which represents an upper limit of the actual receptor cluster size, as the expected cluster size of the receptors is probably below the diffraction limit. To further investigate the clustering of BRI1 and SERK3/BAK1, optical super-resolution techniques, namely photo-activated localization microscopy (PALM) [47], should be employed in combination with VAEM.

It is known that SERK3/BAK1 plays an important role in multiple pathways such as BR signalling and innate immunity (Flagellin) [42, 48]. We hypothesise that each individual nanocluster represents a signalling entity composed of preassembled receptor pairs. Similar clustering has been proposed for the CLAVATA receptor family [49]. The main advantage of preassembly is that the signalling fidelity and response time is improved in comparison to a scenario in which the receptors are homogeneously distributed across the PM, which would require the assembly of signalling units post receptor activation. The nanoclusters would provide spatial separation of receptor pairs and could explain how one protein can play an essential role in multiple signalling pathways.

## Materials & Methods

### Growth conditions and plant lines

*Arabidopsis thaliana* plants of ecotype Columbia (Col-0) were used as wild type. Seeds were surface sterilized and germinated on ½ Murashige and Skoog medium (Duchefa) supplemented with 1% sucrose (Sigma) and 1% Daishin agar (Duchefa). Plants were grown at 22°C under fluorescent light, with 16 h light/8h dark photoperiods. Col-0 plants expressing BRI1 (AT4G39400) fused to GFP under its native promoter, here referred to as BRI1-GFP line 1, were provided by N. Geldner [12]. BRI1-GFP line 2 is a BRI1-GFP line overexpressing the transgene roughly three-fold, and was provided by J. Chory [50]. Col-0 plants expressing SERK3/BAK1-mCherry or SERK3/BAK1-GFP under control of its native promoter were generated as previously described [8]. BRI1-GFP line 2 was crossed with the SERK3-mCherry line to create a plant harbouring both transgenes. S*erk1 serk3* mutant plants harbouring BRI1-GFP was produced by crossing BRI1-GFP line 2 with the double mutant *serk1-3* (GABI-KAT line 448E10) *serk3-2* (SALK_116202) resulting in the *serk1serk3* BRI1-GFP line. The *det*2 seeds were obtained from the *Arabidopsis* seed stock centre and crossed with BRI1-GFP line 2. Col-0 plants containing the transgenes Wave6-mCherry and Wave18-RFP were provided by N. Geldner [26], LT16B-GFP was provided by C. ten Hove [22], VHAa1-mRFP [27], ARA7/Rab F2B-mRFP and ARA6/ Rab F1-mRFP were provided by K. Schumacher, Heidelberg. KNOLLE-GFP was used as a positive control for the FRAP experiments based on the data of [51]. The pBIR3:BIR3-GFP line was constructed by Walter van Dongen (Biochemistry, WU).

### Hormone and inhibitor treatments

For hormone treatment, six-day-old seedlings were incubated in 1 mL ½ Murashige and Skoog medium, supplemented with 1% sucrose and 1 μM 24-epi-brassinolide (BL, Sigma). For brassinazole treatment, seeds were first germinated and grown for four days on ½ Murashige and Skoog medium, supplemented with 1% sucrose and 1% Daishin agar. After four days the seedlings were transferred to plates complemented with 5 μM brassinazole (BRZ, TCI Europe) and grown on these plates for an additional two days.

For FRET-FLIM experiments, 5 day old seedlings were used. The seedlings were transferred to 1 ml ½ MS medium containing 5 μM BRZ 3 days post germination for an additional two days. BR signalling was induced by incubation of seedlings with 1 μM BL for 1 hour.

### Variable-angle epifluorescence microscopy (VAEM)

In total-internal reflection fluorescence microscopy (TIRFM), the laser light is focussed into the rim of the backfocal plane of a microscope objective with high numerical aperture. As a result, the strong inclination of the passing laser light leads to the phenomena of total internal reflection at the interface between the cover slip and the sample medium due to the lower refractive index of the sample medium. Even though the light does not pass the interface, an evanescent wave is generated which decays exponentially within a few hundred nanometers [52]. In animal cells, TIRFM has been used to visualize proteins located in the PM [53–55]. In plant cells, however, utilisation of TIRFM is hampered due to presence of the plant cell wall [56] whose thickness is comparable to the effective excitation depth of the evanescent wave. An alternative to TIRFM is VAEM in which the laser light is focussed closer to the centre of the backfocal plane such that not all light is reflected at the interface between the cover glass and water (buffer); instead, a thin band of illuminating light penetrates the sample allowing for greater penetration depth and yielding a high signal to noise ratio for visualizing biological processes at or near the PM of living cells. By varying the position of the focus in the backfocal plane, we can adjust the depth at which the sample is illuminated. Due to the curvature of the plant root, only a narrow region of the outer PM of the epidermal root cells in close proximity to the cover slip was visualized.

Live root imaging was performed on a home-build microscopy setup described in [57]. The setup is equipped with a 100x/1.49NA TIRF objective (Nikon) and an Ixon Ultra 897 emCCD camera with 512 x 512 pixel (Andor) for imaging. The total magnification of the microscope is 125 x corresponding to a pixel size of 130 nm. Data was recorded using micromanager [58]. GFP was excited with a 473 nm laser (laser power in front of the polychroic mirror 0.98 mW) and fluorescence emission was detected from 480–550 nm. mCherry was excited with a 561 nm laser (laser power set at 0.35 mW) and fluorescence emission was detected from 570–625 nm. Movies containing 250 or 500 frames were recorded every 100 msec, with an exposure time of 100 msec. ImageJ and FIJI were used for data processing (FIJI software, IMAGEJA, 51.45j, Max Planck Society; [59]. For all images of PM localized proteins, a background subtraction (rolling ball radius = 50.0 pixels) was performed. For the analysis of nanoclusters, a Gaussian blur filter of 2 μm (σ) was applied to the fluorescence intensity image. The resulting binary image was thresholded at 80 a.u. and subsequently analysed using the plugin for particle analysis of Image J. For quantification of the number of receptors per nanocluster, we defined areas of 5x5 pixels as region of interest and analysed bleaching decay curves by plotting z-axis profile function.

### Confocal microscopy and FRAP experiments

Roots of *Arabidopsis* seedlings expressing BRI1-GFP line 1, SERK3/BAK1-GFP or KNOLLE-GFP were imaged with a Zeiss CONFOCOR2/LSM510 confocal microscope equipped with a 40x/1.2NA water objective, and an argon laser (output of 6.1 A). For FRAP analysis, the PM was scanned at 488 nm excitation with a laser power of 5% and 9% for BRI1-GFP and SERK3/BAK1-GFP respectively. The fluorescence intensity of GFP was detected with a band-pass filter at 505–550 nm. The image size was set to 512x512 pixels and four scans were averaged for each picture. After 3 scans, a high intensity bleach pulse (50 iterations at 50% laser power) at 488 nm was applied over the selected area. Subsequently, the fluorescence recovery was followed for 499 s and 436 s for BRI1-GFP and SERK3/BAK1-GFP respectively.

FRAP data analysis was performed by subtracting the background signal from the raw data followed by a normalisation of the fluorescence intensity between zero and one (Fig 2 and S8 Fig). The normalised data were plotted using MS Excel, which was also used for curve fitting.

### Selective-surface observation FRET-FLIM

Förster resonance energy transfer (FRET) is a process in which excitation energy is transferred from a donor fluorophore to an acceptor chromophore through nonradiative dipole–dipole coupling [60]. This process can only occur if fluorescent donor and acceptor molecules are at very close proximity. The energy transfer rate is proportional to the inverse 6^th^ power of the distance *R* between donor and acceptor, which makes this method extremely sensitive for distances at protein level dimensions (<10 nm). FRET determined using fluorescence lifetime imaging microscopy (FLIM) is independent of protein concentration, but very sensitive for the local microenvironment of the fluorophores. In FRET-FLIM, the fluorescence lifetime of the donor molecule is reduced in presence of an acceptor molecule nearby since energy transfer to the acceptor will introduce an additional relaxation path from the excited to the ground state of the donor. The FRET efficiency (E) is given by E = (1 - *τ*_DA_/ *τ*_D_) where *τ*_DA_ is the fluorescence lifetime of the donor in the presence of acceptor and *τ*_D_ is the fluorescence lifetime of the donor alone.

Selective-surface observation (SSO)-FRET-FLIM was performed on a Leica TCS SP5 X equipped with a 63X/1.2NA water immersion objective. In SSO-FRET-FLIM, the confocal spot is positioned perpendicular to the PM of the root epidermal cells. In this configuration, it was possible to observe signals from the PM whilst largely omitting signals from underlying organelles such as the cortical ER. A 40 MHz tunable supercontinuum laser was used to excite GFP and mCherry at 488 nm and 587 nm, respectively. Fluorescence emission was detected using an internal Hybrid (HyD) detector with 100 ps time resolution and collected in a spectral window of 495–550 nm for GFP and 500–540 nm for mCherry provided by an Acousto-Optical Beam Splitter. The signal output from the HyD was coupled to an external time-correlated single photon counting module (Becker&Hickl) for acquiring FLIM data. Typical images had 128 x 128 pixels (pixel size ± 300 nm), and 256 time channels per pixel with an acquisition time of 90–120 seconds per image.

From the time resolved fluorescence intensity images, the fluorescence decay curves were calculated for each pixel and fitted with either a mono- or double-exponential decay model using the SPCImage v5.0 software (Becker & Hickl). Fitting was performed without fixing any parameters. FRET-FLIM analysis provided fluorescence intensity as well as false-colored fluorescence lifetime images. The raw data was subjected to the following criteria to analyze and omit false positive negatives in the fluorescence lifetime scoring: minimum photon count per pixel of 1200 photons, goodness of fit (χ^2^<2) and fluorescence lifetime range of 1500–2500 ps. For data analysis, we set pixel binning at 1 to have sufficient number of photons per pixel required for accurate fluorescence lifetime analysis.

In addition, a numerical evaluation of the observed fluorescence lifetime values and interaction pixels (IPS) was determined by exporting the fitted data from SPCImage into a Phyton written script, which calculates the number of pixels that adheres to the above-mentioned fitting criteria. The fraction of IPS was established by applying a fluorescence lifetime threshold as a percentage of the total number of pixels [8]. This threshold, corresponding to a FRET efficiency of about 13%, was used and only pixels with fluorescence lifetimes below this interaction threshold were collected as IPS.

### Accession numbers

Sequence data from this article can be found in the *Arabidopsis* Genome Initiative or EMBL/GenBank data libraries under accession numbers: BRI1 (AT4G39400), SERK3/BAK1 (AT4G22430), WAVE6/NIP1;1 (AT4G19030), WAVE18/Got1p homolog (AT3G03180), ARA6/RABF1 (AT3G54840), ARA7/RABF2B (AT4G19640), VHA-A1 (AT2G28520) LTI6B/RCI2B (AT3G05890), DET2 (AT2G38050) KNOLLE (AT1G08560) and BIR3 (AT1G27190).

## Supporting Information

S1 Fig Visualization of A. thaliana membrane compartments with VAEM. Live-cell VAEM imaging performed on 6 day old Arabidopsis seedling roots expressing fluorescent markers for different membrane compartments. (A) PM localized LT16B-GFP, (B) ER localized WAVE6-mCherry, (C) Golgi localized WAVE18-mRFP, (D) TGN localized VHAa1-mRFP, (E) EE/LE localized ARA7-mRFP and (F) LE localized ARA6/Rab F1-mRFP. The exposure time for all images was 100 msec except for B in which 40 sequential images of 100 msec each were combined. Scale bars represent 10 μm.
(TIF)

S2 Fig ImageJ processing for nanocluster analysis. VAEM images of cluster forming BRI1-GFP (**A**) and the PM marker LT16B-GFP (**B**) were analysed. The respective original image was processed by application of a Gaussian blur filter with of 2 μm (σ) followed by subtraction of the blurred image from the original image. Subsequently, a threshold of 80 a.u. and a black-white inversion was applied. (**A**) Nanocluster analysis was performed only in regions within each cell (here exemplified by an ROI marked in green). For further details on the cluster analysis please see the materials and methods section. (**B**) LT16B-GFP does not show the formation of nanoclusters. In fact, only single, non-connected pixels appear in the final image.
(TIF)

S3 Fig VAEM fluorescence decay curves. Shown are two representative decay curves of SERK/BAK1-mCherry clusters, and of BRI1-GFP line 1 clusters. As can be seen, the decay curves of SERK3-mCherry sometimes portrays almost a single molecule behaviour, but at other times, more receptors are present in a cluster. For both receptors, discreet decrease in fluorescence is observed, indicating that the number of receptors in the cluster must be limited.
(TIF)

S4 Fig PM Receptor distribution of PIN2-GFP, BIR3-GFP, BRI1-GFP line 1 and Col0 *Arabidopsis thaliana* in live epidermal root cells using VAEM. Live-cell VAEM imaging performed on 6 day old *Arabidopsis* seedling roots expressing **(A)** PM localized PIN2-GFP, **(B)** PM localized BIR3-GFP, **(C)** PM localized BRI1-GFP line 1, **(D)** *Arabidopsis thaliana* ecotype Columbia.
(TIF)

S5 Fig VAEM images of BRZ treated BRI1-GFP Line 2 epidermal root cells upon BL stimulation. Live-cell VAEM imaging performed on 6 day old *Arabidopsis* seedling roots expressing BRI1-GFP **(A)** PM localized BRI1-GFP, **(B)** PM localized BRI1-GFP treated with 5 μM brassinazole for 3 days, **(C)** PM localized BRI1-GFP treated with 5 μM brassinazole for 3 days and subsequently with 1 μM 24-epi-brassinolide for 1 h, **(D)** PM distribution of BRI1-GFP in the *det2* BR biosynthesis mutant.
(TIF)

S6 Fig Example of typical FRAP experiment. In Fig S6 A and B, images of BRI1-GFP at different scanning iterations are shown. (**A**) shows images that undergo only scan bleaching whereas (**B**) contains features of FRAP region convoluted with scan bleaching. (**C**) Plots of the fluorescence intensity versus number of scans (top orange line: ROI in (A), middle grey line: ROI in (B), blue line: background intensity). (**D**) Normalised FRAP curve, corrected for scan bleaching. As shown, BRI1-GFP receptors are largely immobile. Furthermore, scan bleaching strongly interferes with the interpretation of the dynamics of the recovery.
(TIF)

S7 Fig Gaussian fits of receptor molecules in PM surrounding the anticlinal cell wall before and after photobleaching. The line represents the fit of a Gaussian distribution on the fluorescence intensity data across the anticlinal cell wall. Distance 0 is the midpoint of two adjacent plasma membranes in a confocal image. The cytoplasm is situated between 1–0,5 μm on either side of the midpoint. **(A)** Fluorescence intensity of BRI1-GFP at the bleached area (left) compared to the intensity at a non-bleached area of the PM (right). After 400 seconds, the fluorescence intensity at the non-bleached area (right panel) was reduced significantly due to scan bleaching. **(B)** Same as A, except now for SERK3/BAK1-GFP. n = 5 different roots; 20 fits per image (n ≥100 data points).
(TIF)

S8 Fig FRAP data analysis. (TIF)

S1 Movie Dynamics of ER marker (VMA21-GFP). (AVI)

S2 Movie Movie of BRI1-GFP in presence of BL. (AVI)

S3 Movie Movie of BRI1-GFP in presence of BL. (AVI)

S4 Movie Movie of BRI1-GFP in absence of BL. (AVI)

S5 Movie Movie of SERK3-GFP. (AVI)

S6 Movie Movie of SERK3-GFP. (AVI)
