# Supplementary figures and images for: Visualization of BRI1 and SERK3/BAK1 Nanoclusters in Arabidopsis Roots
Source: PLoS One. 2017 Jan 23;12(1):e0169905. doi: 10.1371/journal.pone.0169905 (PMC5256950; doi:10.1371/journal.pone.0169905)

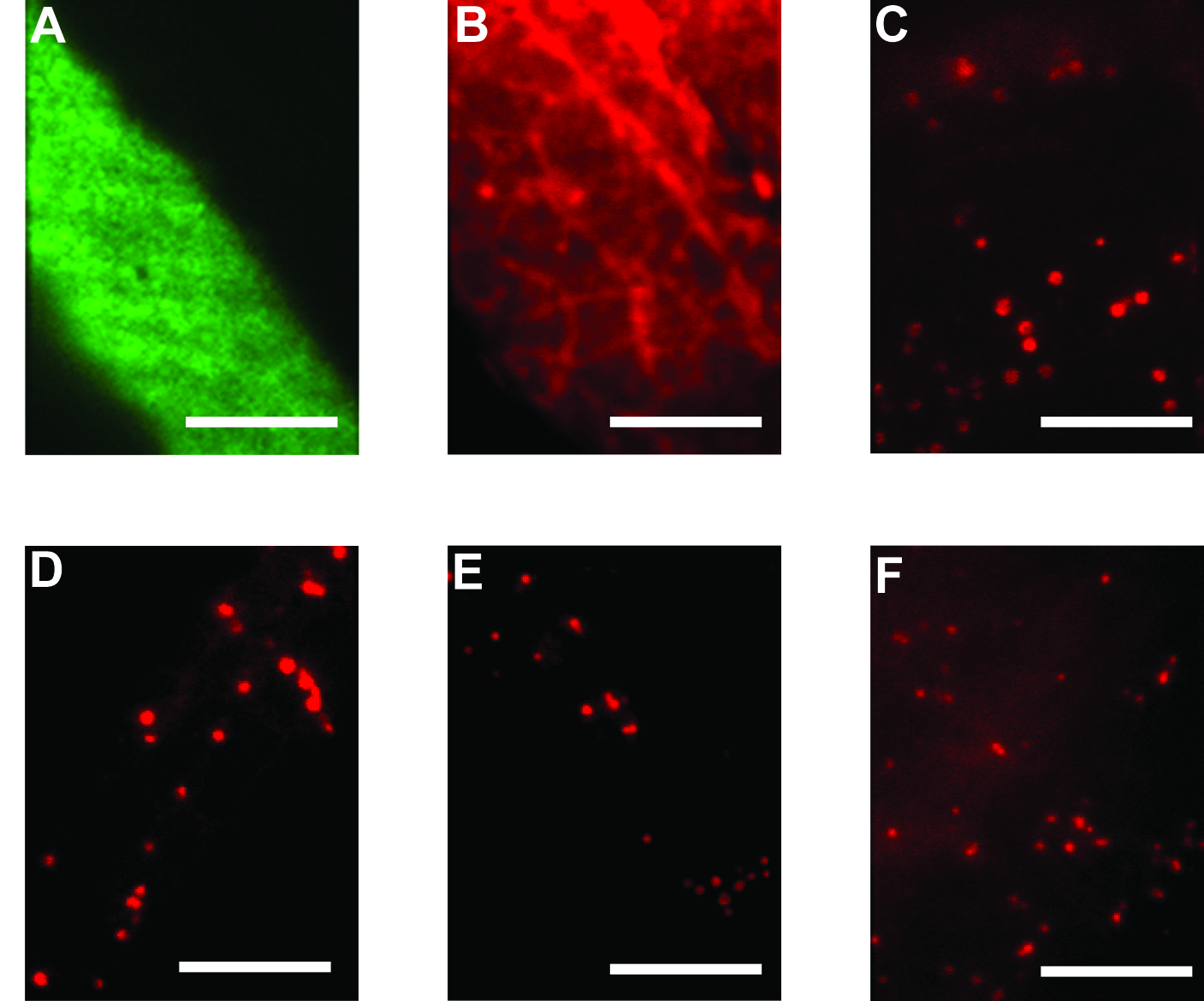

Supplement: S1 Fig — Live-cell VAEM imaging performed on 6 day old Arabidopsis seedling roots expressing fluorescent markers for different membrane compartments. (A) PM localized LT16B-GFP, (B) ER localized WAVE6-mCherry, (C) Golgi localized WAVE18-mRFP, (D) TGN localized VHAa1-mRFP, (E) EE/LE localized ARA7-mRFP and (F) LE localized ARA6/Rab F1-mRFP. The exposure time for all images was 100 msec except for B in which 40 sequential images of 100 msec each were combined. Scale bars represent 10 μm. (TIF) [file pone.0169905.s001.tif]

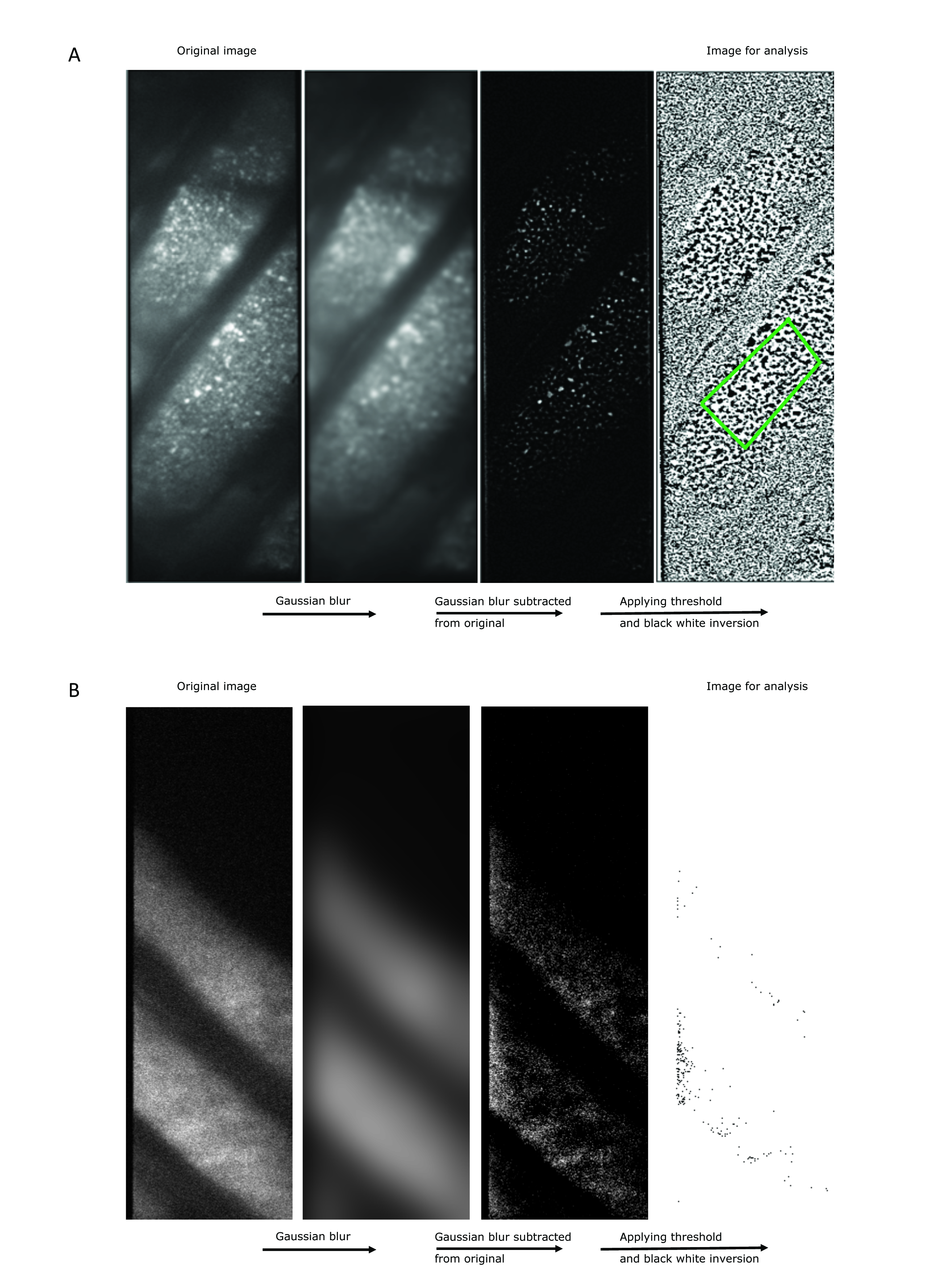

Supplement: S2 Fig — VAEM images of cluster forming BRI1-GFP (A) and the PM marker LT16B-GFP (B) were analysed. The respective original image was processed by application of a Gaussian blur filter with of 2 μm (σ) followed by subtraction of the blurred image from the original image. Subsequently, a threshold of 80 a.u. and a black-white inversion was applied. (A) Nanocluster analysis was performed only in regions within each cell (here exemplified by an ROI marked in green). For further details on the cluster analysis please see the materials and methods section. (B) LT16B-GFP does not show the formation of nanoclusters. In fact, only single, non-connected pixels appear in the final image. (TIF) [file pone.0169905.s002.tif]

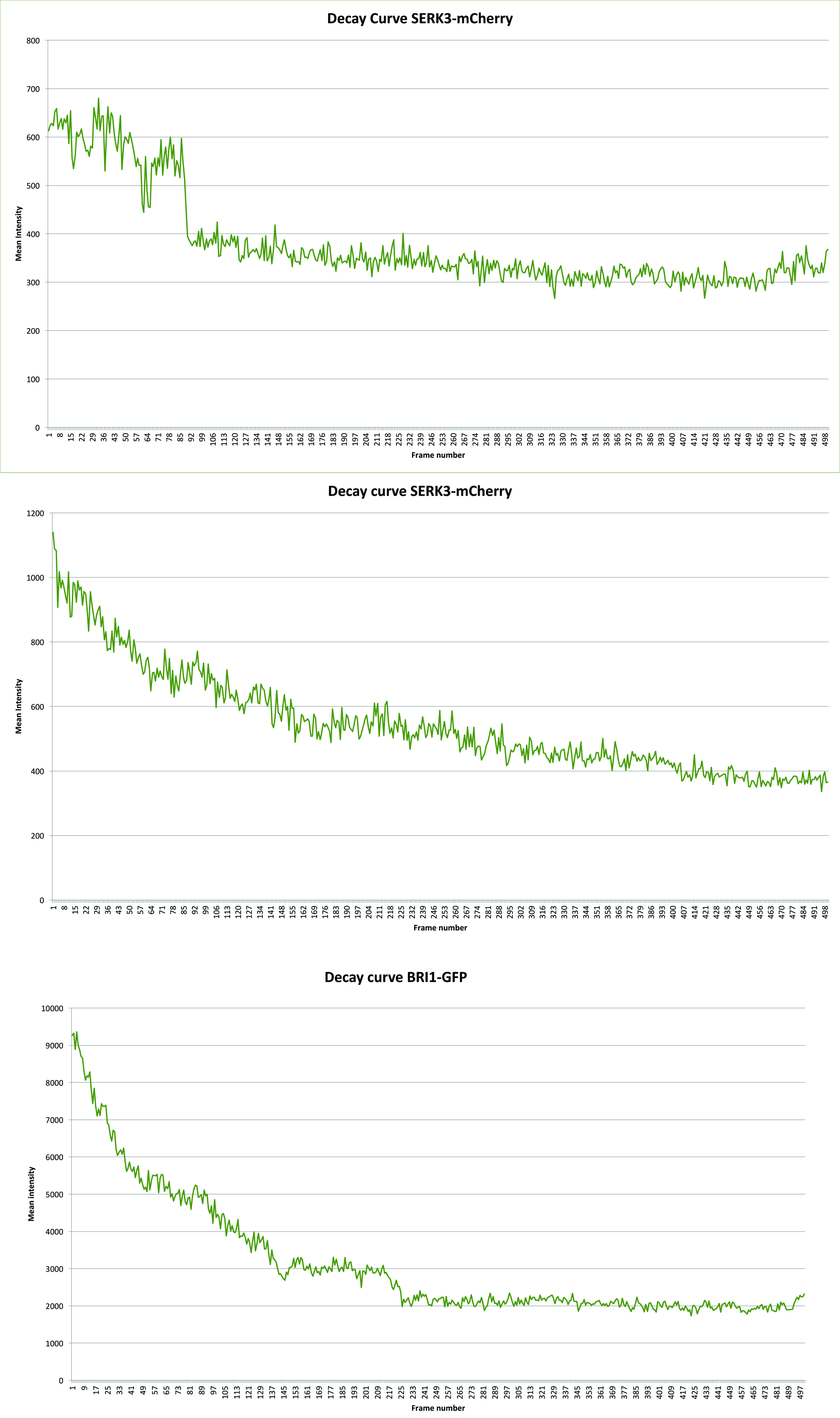

Supplement: S3 Fig — Shown are two representative decay curves of SERK/BAK1-mCherry clusters, and of BRI1-GFP line 1 clusters. As can be seen, the decay curves of SERK3-mCherry sometimes portrays almost a single molecule behaviour, but at other times, more receptors are present in a cluster. For both receptors, discreet decrease in fluorescence is observed, indicating that the number of receptors in the cluster must be limited. (TIF) [file pone.0169905.s003.tif]

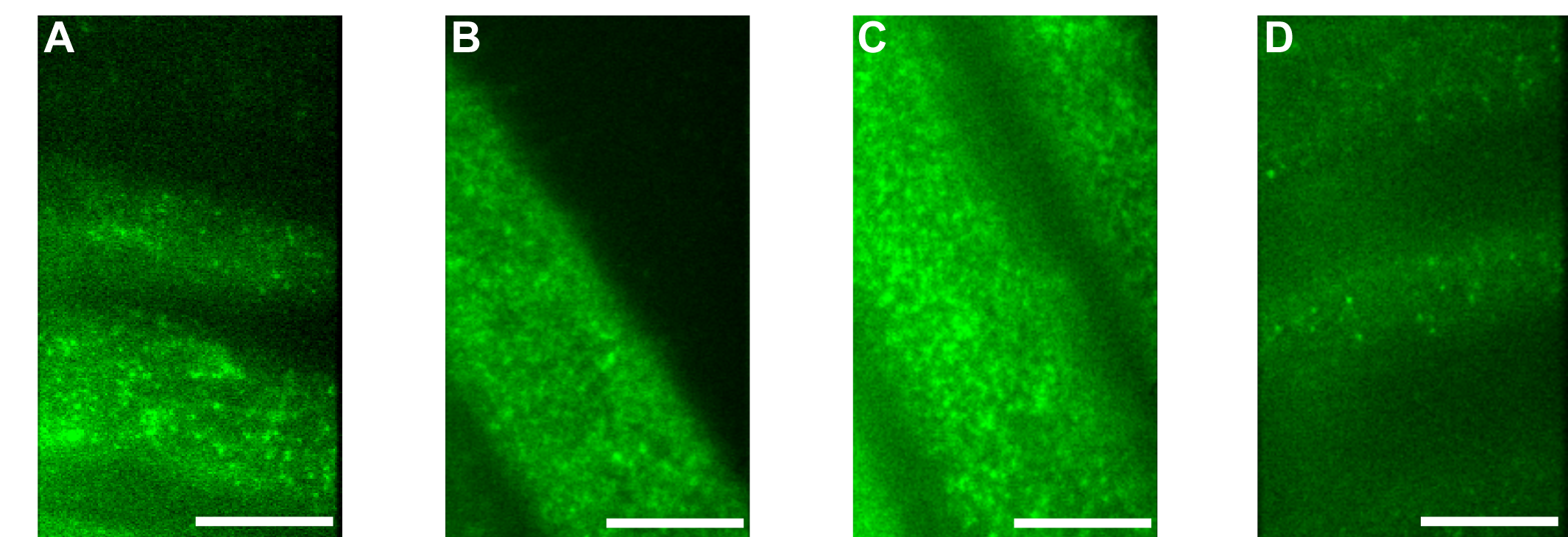

Supplement: S4 Fig — Live-cell VAEM imaging performed on 6 day old Arabidopsis seedling roots expressing (A) PM localized PIN2-GFP, (B) PM localized BIR3-GFP, (C) PM localized BRI1-GFP line 1, (D) Arabidopsis thaliana ecotype Columbia. (TIF) [file pone.0169905.s004.tif]

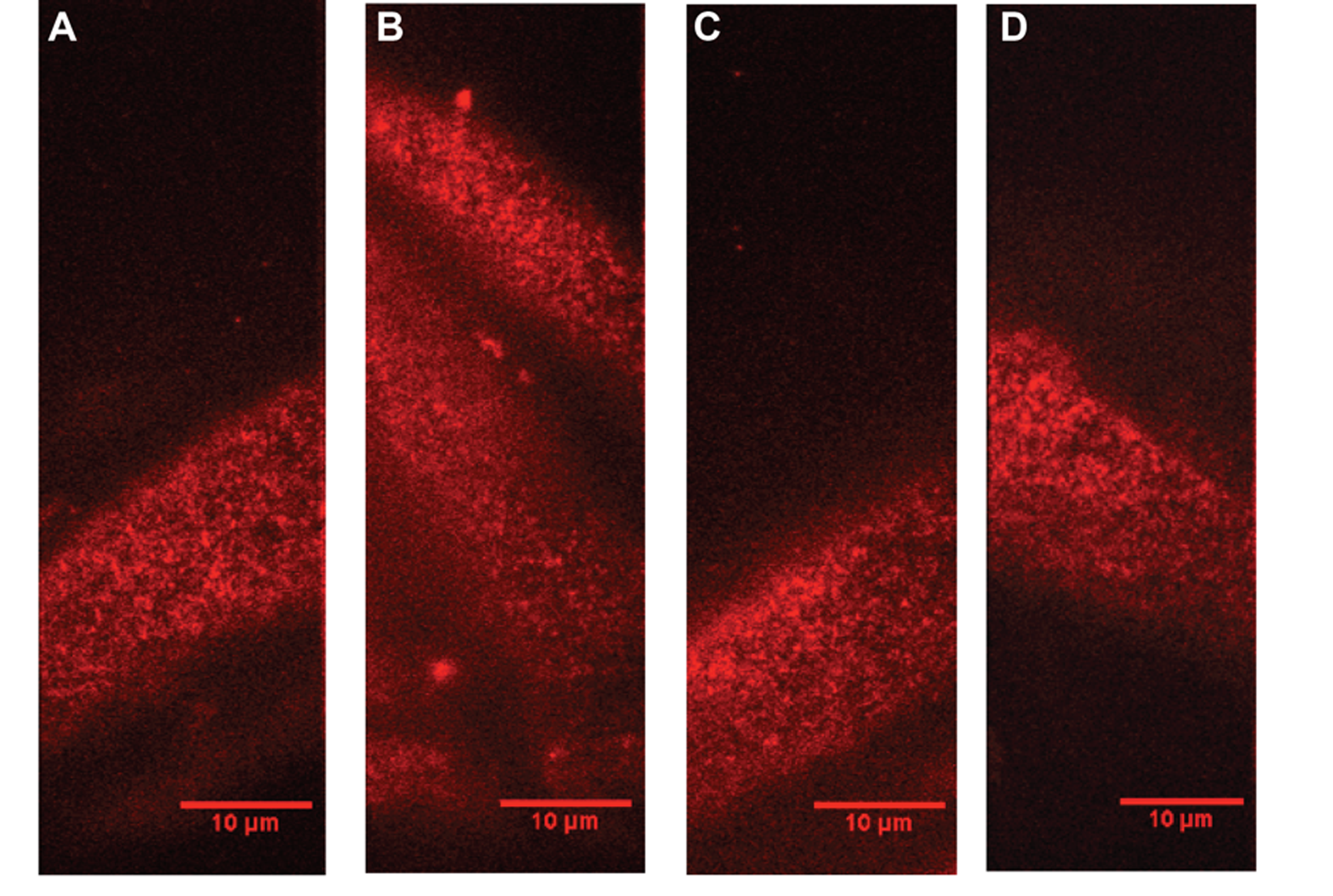

Supplement: S5 Fig — Live-cell VAEM imaging performed on 6 day old Arabidopsis seedling roots expressing BRI1-GFP (A) PM localized BRI1-GFP, (B) PM localized BRI1-GFP treated with 5 μM brassinazole for 3 days, (C) PM localized BRI1-GFP treated with 5 μM brassinazole for 3 days and subsequently with 1 μM 24-epi-brassinolide for 1 h, (D) PM distribution of BRI1-GFP in the det2 BR biosynthesis mutant. (TIF) [file pone.0169905.s005.tif]

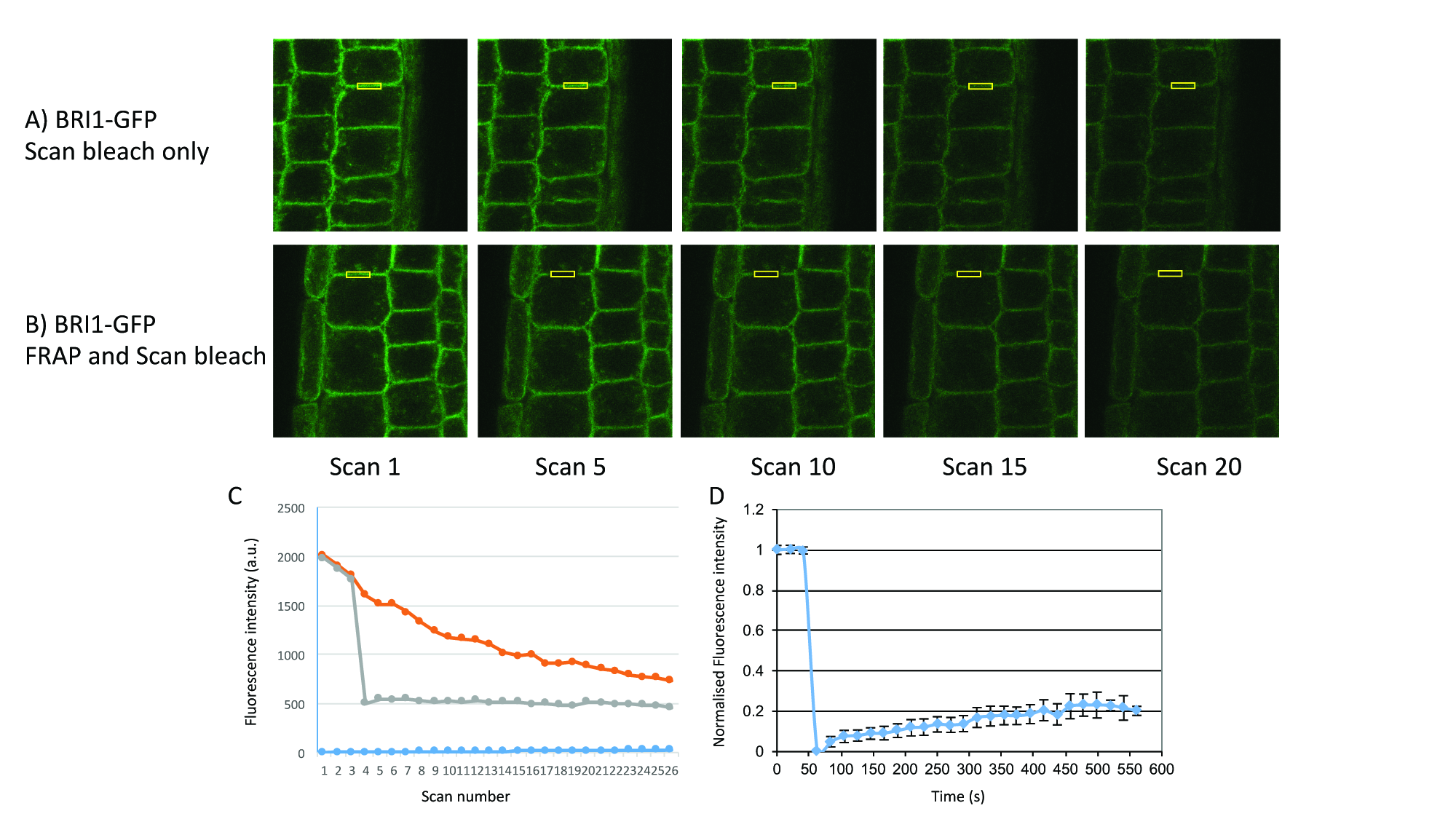

Supplement: S6 Fig — In Fig S6 A and B, images of BRI1-GFP at different scanning iterations are shown. (A) shows images that undergo only scan bleaching whereas (B) contains features of FRAP region convoluted with scan bleaching. (C) Plots of the fluorescence intensity versus number of scans (top orange line: ROI in (A), middle grey line: ROI in (B), blue line: background intensity). (D) Normalised FRAP curve, corrected for scan bleaching. As shown, BRI1-GFP receptors are largely immobile. Furthermore, scan bleaching strongly interferes with the interpretation of the dynamics of the recovery. (TIF) [file pone.0169905.s006.tif]

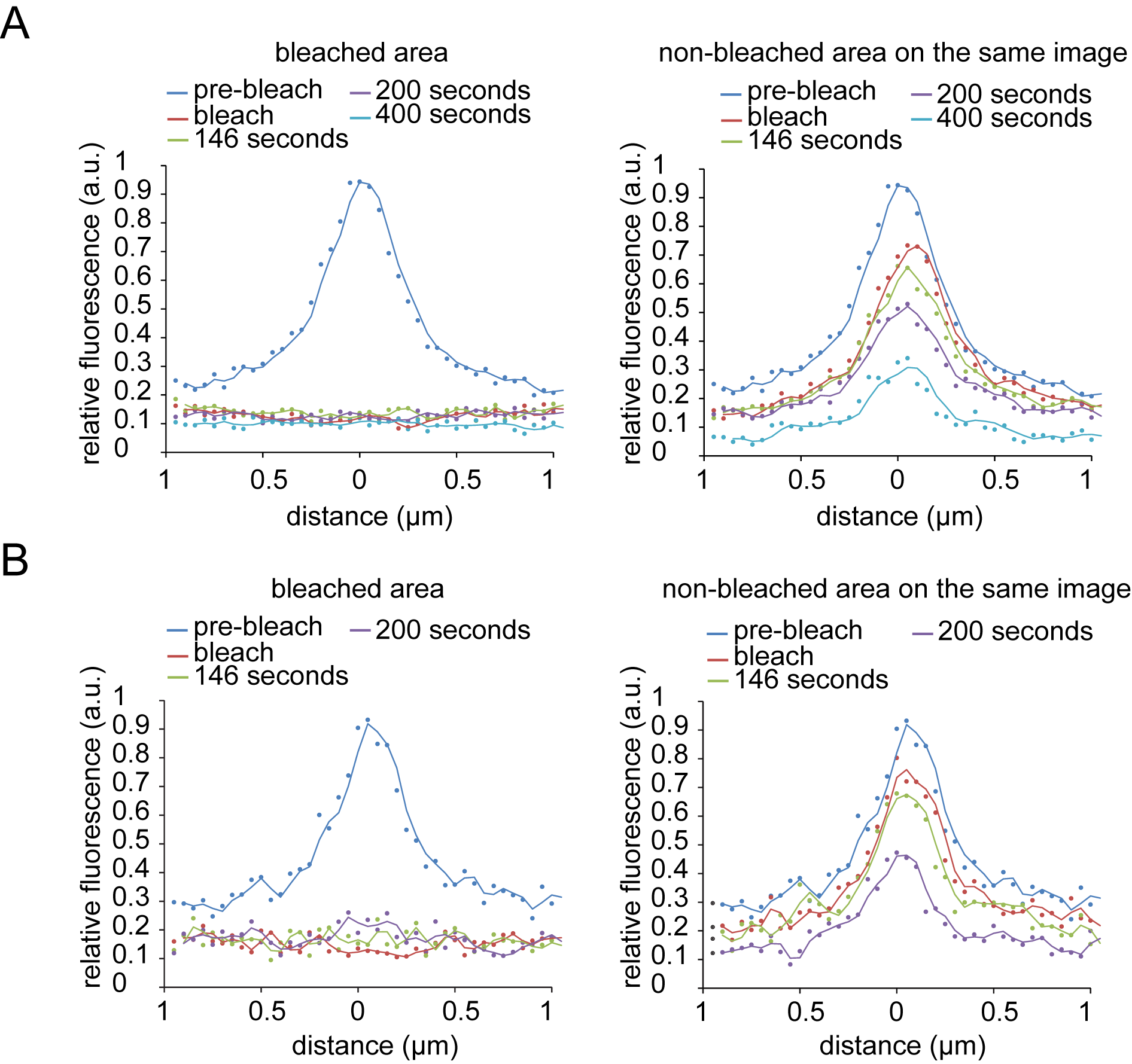

Supplement: S7 Fig — The line represents the fit of a Gaussian distribution on the fluorescence intensity data across the anticlinal cell wall. Distance 0 is the midpoint of two adjacent plasma membranes in a confocal image. The cytoplasm is situated between 1–0,5 μm on either side of the midpoint. (A) Fluorescence intensity of BRI1-GFP at the bleached area (left) compared to the intensity at a non-bleached area of the PM (right). After 400 seconds, the fluorescence intensity at the non-bleached area (right panel) was reduced significantly due to scan bleaching. (B) Same as A, except now for SERK3/BAK1-GFP. n = 5 different roots; 20 fits per image (n ≥100 data points). (TIF) [file pone.0169905.s007.tif]

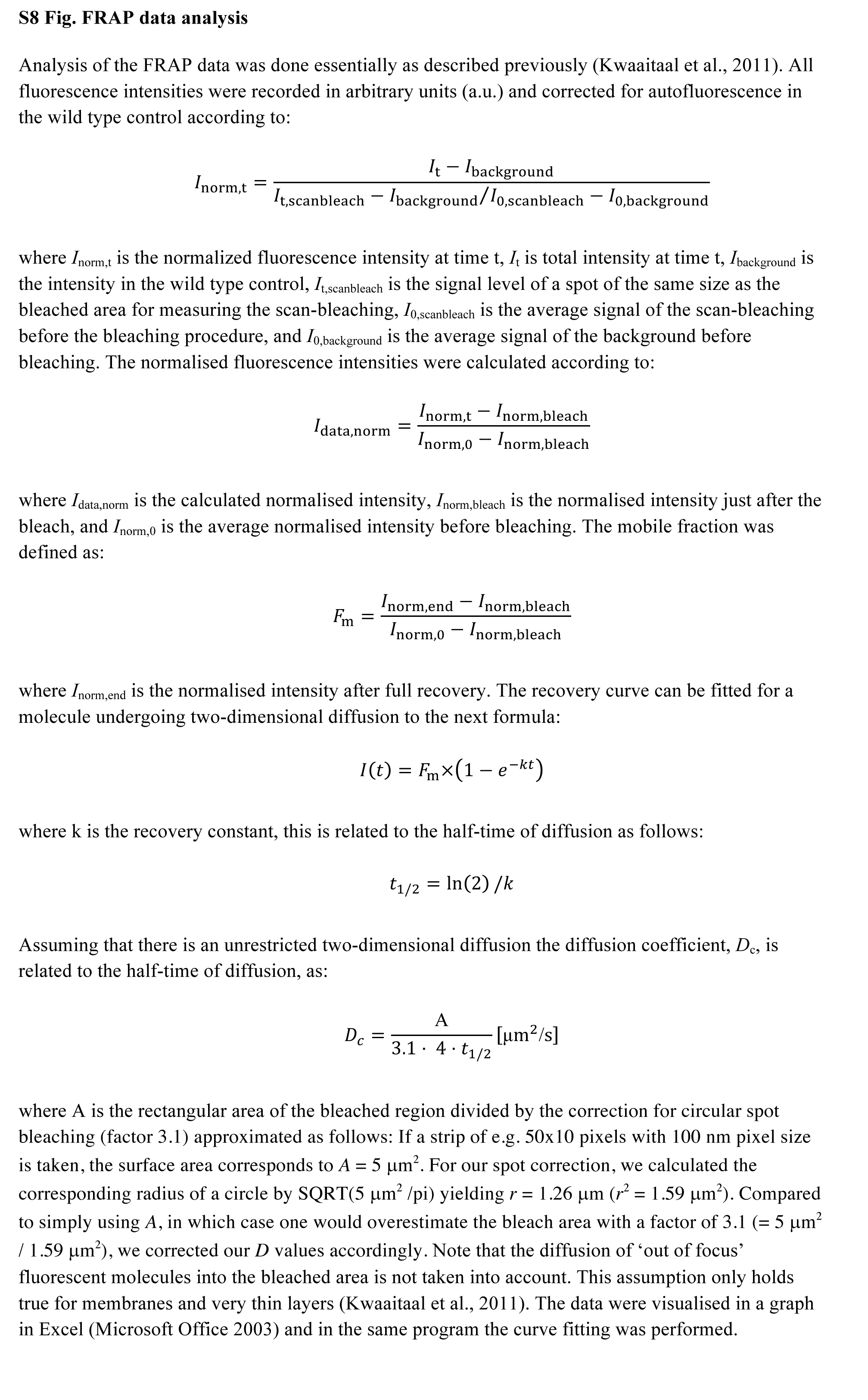

Supplement: S8 Fig — (TIF) [file pone.0169905.s008.tif]
